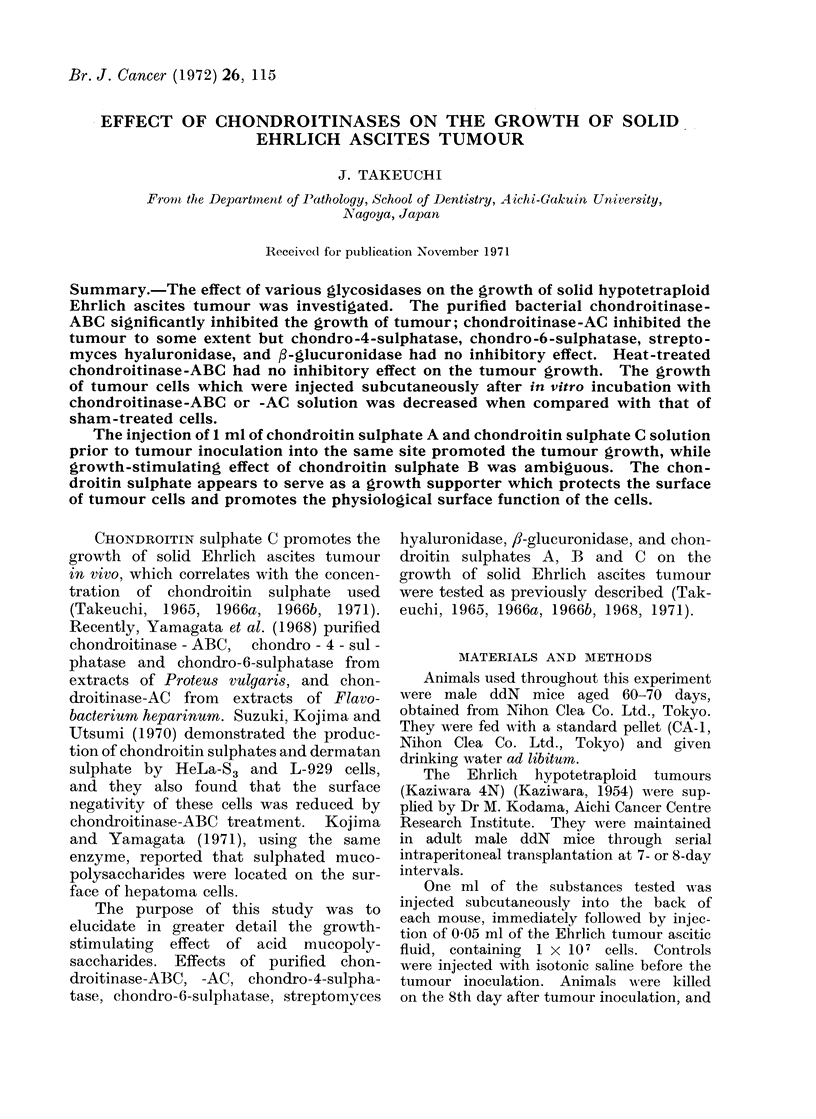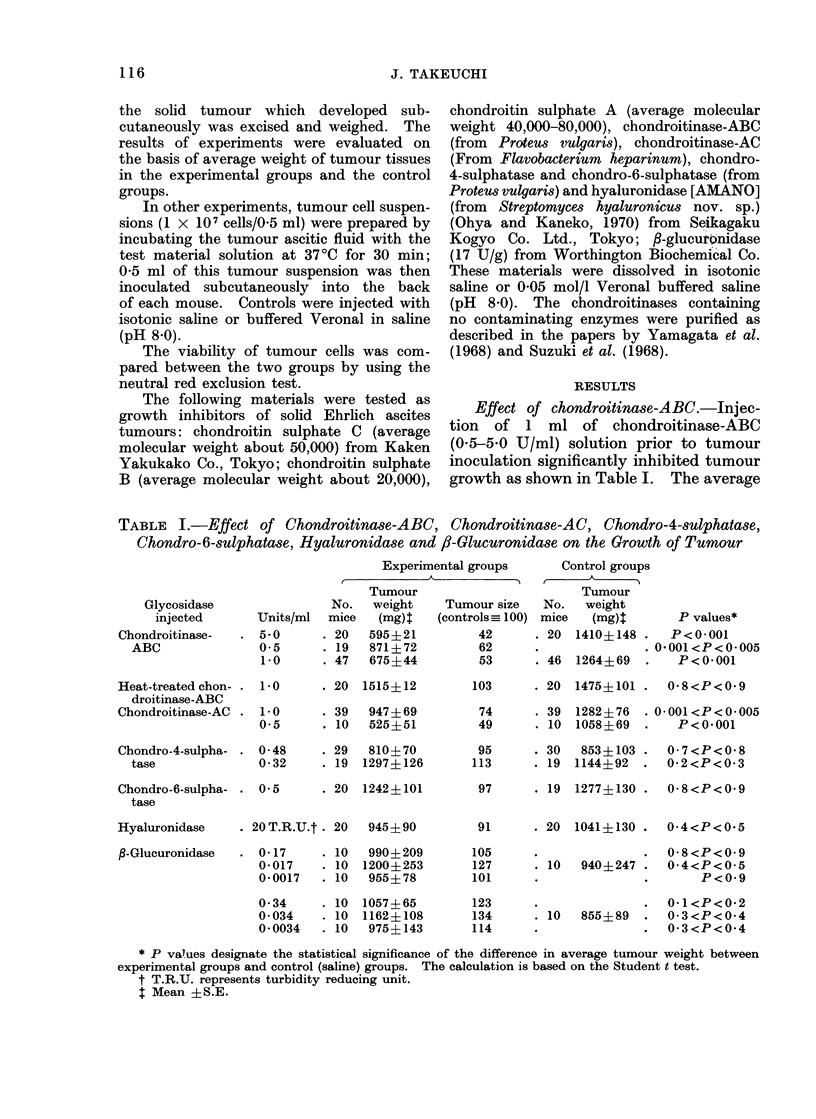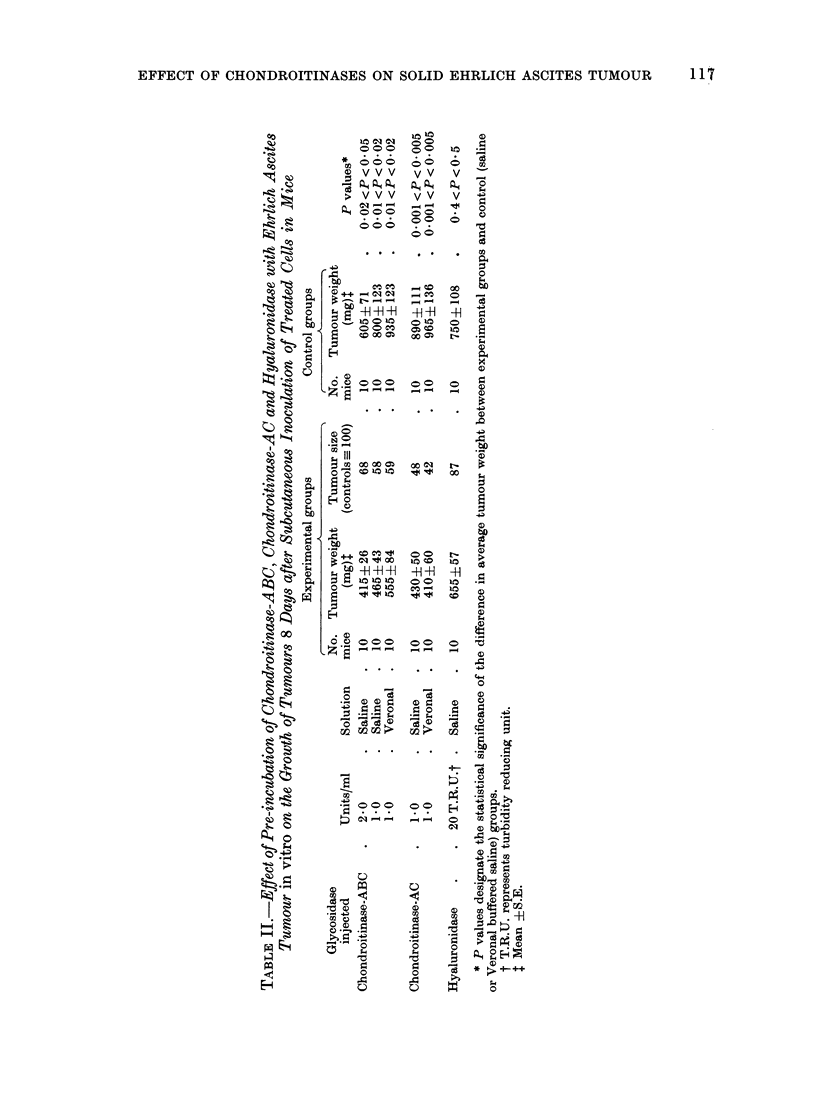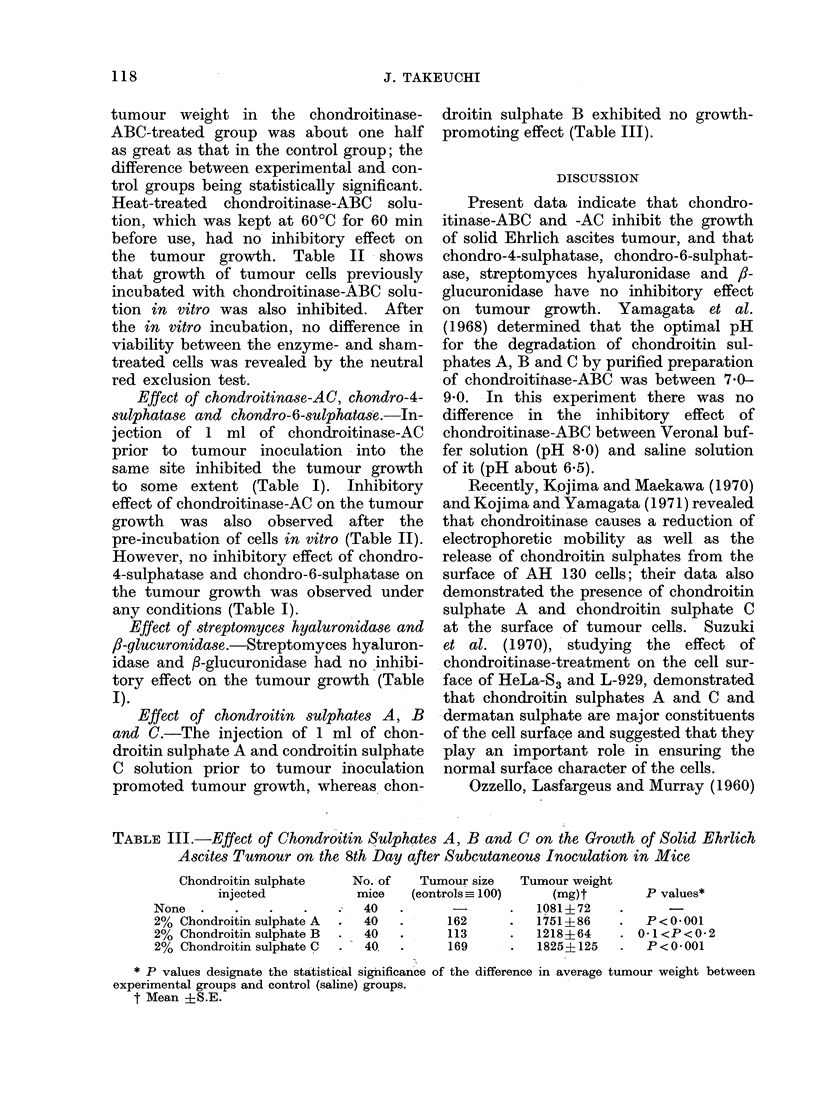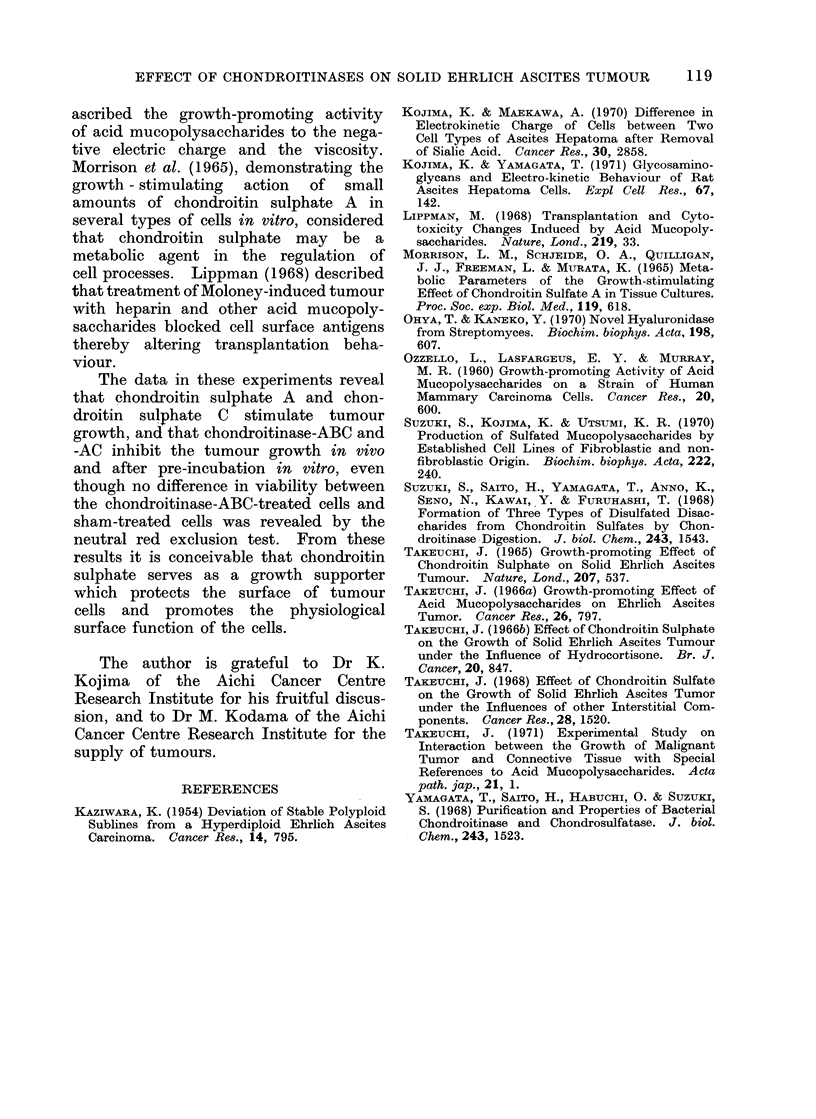# Effect of Chondroitinases on the Growth of Solid Ehrlich Ascites Tumour

**DOI:** 10.1038/bjc.1972.17

**Published:** 1972-04

**Authors:** J. Takeuchi

## Abstract

The effect of various glycosidases on the growth of solid hypotetraploid Ehrlich ascites tumour was investigated. The purified bacterial chondroitinase-ABC significantly inhibited the growth of tumour; chondroitinase-AC inhibited the tumour to some extent but chondro-4-sulphatase, chondro-6-sulphatase, streptomyces hyaluronidase, and β-glucuronidase had no inhibitory effect. Heat-treated chondroitinase-ABC had no inhibitory effect on the tumour growth. The growth of tumour cells which were injected subcutaneously after *in vitro* incubation with chondroitinase-ABC or -AC solution was decreased when compared with that of sham-treated cells.

The injection of 1 ml of chondroitin sulphate A and chondroitin sulphate C solution prior to tumour inoculation into the same site promoted the tumour growth, while growth-stimulating effect of chondroitin sulphate B was ambiguous. The chondroitin sulphate appears to serve as a growth supporter which protects the surface of tumour cells and promotes the physiological surface function of the cells.


*Br. J. Cancer* (1972) **26**, 115

### EFFECT OF CHONDROITINASES ON THE GROWTH OF SOLID EHRLICH ASCITES TUMOUR

J. TAKEUCHI

*From the Department of Pathology, School of Dentistry, Aichi-Gakuin University, Nagoya, Japan*

Received for publication November 1971

**Summary.—The effect of various glycosidases on the growth of solid hypotetraploid Ehrlich ascites tumour was investigated. The purified bacterial chondroitinase-ABC significantly inhibited the growth of tumour; chondroitinase-AC inhibited the tumour to some extent but chondro-4-sulphatase, chondro-6-sulphatase, streptomyces hyaluronidase, and β-glucuronidase had no inhibitory effect. Heat-treated chondroitinase-ABC had no inhibitory effect on the tumour growth. The growth of tumour cells which were injected subcutaneously after *in vitro* incubation with chondroitinase-ABC or -AC solution was decreased when compared with that of sham-treated cells.**

**The injection of 1 ml of chondroitin sulphate A and chondroitin sulphate C solution prior to tumour inoculation into the same site promoted the tumour growth, while growth-stimulating effect of chondroitin sulphate B was ambiguous. The chondroitin sulphate appears to serve as a growth supporter which protects the surface of tumour cells and promotes the physiological surface function of the cells.**

CHONDROITIN sulphate C promotes the growth of solid Ehrlich ascites tumour *in vivo*, which correlates with the concentration of chondroitin sulphate used (Takeuchi, 1965, 1966*a*, 1966*b*, 1971). Recently, Yamagata *et al.* (1968) purified chondroitinase - ABC, chondro - 4 - sulphatase and chondro-6-sulphatase from extracts of *Proteus vulgaris*, and chondroitinase-AC from extracts of *Flavobacterium heparinum*. Suzuki, Kojima and Utsumi (1970) demonstrated the production of chondroitin sulphates and dermatan sulphate by HeLa-$S_3$ and L-929 cells, and they also found that the surface negativity of these cells was reduced by chondroitinase-ABC treatment. Kojima and Yamagata (1971), using the same enzyme, reported that sulphated mucopolysaccharides were located on the surface of hepatoma cells.

The purpose of this study was to elucidate in greater detail the growth-stimulating effect of acid mucopolysaccharides. Effects of purified chondroitinase-ABC, -AC, chondro-4-sulphatase, chondro-6-sulphatase, streptomyces hyaluronidase, β-glucuronidase, and chondroitin sulphates A, B and C on the growth of solid Ehrlich ascites tumour were tested as previously described (Takeuchi, 1965, 1966*a*, 1966*b*, 1968, 1971).

### MATERIALS AND METHODS

Animals used throughout this experiment were male ddN mice aged 60–70 days, obtained from Nihon Clea Co. Ltd., Tokyo. They were fed with a standard pellet (CA-1, Nihon Clea Co. Ltd., Tokyo) and given drinking water *ad libitum*.

The Ehrlich hypotetraploid tumours (Kaziwara 4N) (Kaziwara, 1954) were supplied by Dr M. Kodama, Aichi Cancer Centre Research Institute. They were maintained in adult male ddN mice through serial intraperitoneal transplantation at 7- or 8-day intervals.

One ml of the substances tested was injected subcutaneously into the back of each mouse, immediately followed by injection of 0·05 ml of the Ehrlich tumour ascitic fluid, containing $1 \times 10^7$ cells. Controls were injected with isotonic saline before the tumour inoculation. Animals were killed on the 8th day after tumour inoculation, and the solid tumour which developed subcutaneously was excised and weighed. The results of experiments were evaluated on the basis of average weight of tumour tissues in the experimental groups and the control groups.

In other experiments, tumour cell suspensions ($1 \times 10^7$ cells/0·5 ml) were prepared by incubating the tumour ascitic fluid with the test material solution at 37°C for 30 min; 0·5 ml of this tumour suspension was then inoculated subcutaneously into the back of each mouse. Controls were injected with isotonic saline or buffered Veronal in saline (pH 8·0).

The viability of tumour cells was compared between the two groups by using the neutral red exclusion test.

The following materials were tested as growth inhibitors of solid Ehrlich ascites tumours: chondroitin sulphate C (average molecular weight about 50,000) from Kaken Yakukako Co., Tokyo; chondroitin sulphate B (average molecular weight about 20,000), chondroitin sulphate A (average molecular weight 40,000–80,000), chondroitinase-ABC (from *Proteus vulgaris*), chondroitinase-AC (From *Flavobacterium heparinum*), chondro-4-sulphatase and chondro-6-sulphatase (from *Proteus vulgaris*) and hyaluronidase [AMANO] (from *Streptomyces hyaluronicus* nov. sp.) (Ohya and Kaneko, 1970) from Seikagaku Kogyo Co. Ltd., Tokyo; β-glucuronidase (17 U/g) from Worthington Biochemical Co. These materials were dissolved in isotonic saline or 0·05 mol/l Veronal buffered saline (pH 8·0). The chondroitinases containing no contaminating enzymes were purified as described in the papers by Yamagata *et al.* (1968) and Suzuki *et al.* (1968).

### RESULTS

*Effect of chondroitinase-ABC.*—Injection of 1 ml of chondroitinase-ABC (0·5–5·0 U/ml) solution prior to tumour inoculation significantly inhibited tumour growth as shown in Table I. The average

TABLE I.—*Effect of Chondroitinase-ABC, Chondroitinase-AC, Chondro-4-sulphatase, Chondro-6-sulphatase, Hyaluronidase and β-Glucuronidase on the Growth of Tumour*

| | | Experimental groups | | | Control groups | | |
|---|---|---|---|---|---|---|---|
| Glycosidase injected | Units/ml | No. mice | Tumour weight (mg)‡ | Tumour size (controls ≡ 100) | No. mice | Tumour weight (mg)‡ | *P* values* |
| Chondroitinase-ABC | 5·0 | 20 | 595±21 | 42 | 20 | 1410±148 | $P<0\cdot001$ |
| | 0·5 | 19 | 871±72 | 62 | | | $0\cdot001<P<0\cdot005$ |
| | 1·0 | 47 | 675±44 | 53 | 46 | 1264±69 | $P<0\cdot001$ |
| Heat-treated chondroitinase-ABC | 1·0 | 20 | 1515±12 | 103 | 20 | 1475±101 | $0\cdot8<P<0\cdot9$ |
| Chondroitinase-AC | 1·0 | 39 | 947±69 | 74 | 39 | 1282±76 | $0\cdot001<P<0\cdot005$ |
| | 0·5 | 10 | 525±51 | 49 | 10 | 1058±69 | $P<0\cdot001$ |
| Chondro-4-sulphatase | 0·48 | 29 | 810±70 | 95 | 30 | 853±103 | $0\cdot7<P<0\cdot8$ |
| | 0·32 | 19 | 1297±126 | 113 | 19 | 1144±92 | $0\cdot2<P<0\cdot3$ |
| Chondro-6-sulphatase | 0·5 | 20 | 1242±101 | 97 | 19 | 1277±130 | $0\cdot8<P<0\cdot9$ |
| Hyaluronidase | 20 T.R.U.† | 20 | 945±90 | 91 | 20 | 1041±130 | $0\cdot4<P<0\cdot5$ |
| β-Glucuronidase | 0·17 | 10 | 990±209 | 105 | | | $0\cdot8<P<0\cdot9$ |
| | 0·017 | 10 | 1200±253 | 127 | 10 | 940±247 | $0\cdot4<P<0\cdot5$ |
| | 0·0017 | 10 | 955±78 | 101 | | | $P<0\cdot9$ |
| | 0·34 | 10 | 1057±65 | 123 | | | $0\cdot1<P<0\cdot2$ |
| | 0·034 | 10 | 1162±108 | 134 | 10 | 855±89 | $0\cdot3<P<0\cdot4$ |
| | 0·0034 | 10 | 975±143 | 114 | | | $0\cdot3<P<0\cdot4$ |

* *P* values designate the statistical significance of the difference in average tumour weight between experimental groups and control (saline) groups. The calculation is based on the Student *t* test.

† T.R.U. represents turbidity reducing unit.

‡ Mean ±S.E.

‡ Mean ±S.E.

tumour weight in the chondroitinase-ABC-treated group was about one half as great as that in the control group; the difference between experimental and control groups being statistically significant. Heat-treated chondroitinase-ABC solution, which was kept at 60°C for 60 min before use, had no inhibitory effect on the tumour growth. Table II shows that growth of tumour cells previously incubated with chondroitinase-ABC solution *in vitro* was also inhibited. After the *in vitro* incubation, no difference in viability between the enzyme- and sham-treated cells was revealed by the neutral red exclusion test.

*Effect of chondroitinase-AC, chondro-4-sulphatase and chondro-6-sulphatase.*—Injection of 1 ml of chondroitinase-AC prior to tumour inoculation into the same site inhibited the tumour growth to some extent (Table I). Inhibitory effect of chondroitinase-AC on the tumour growth was also observed after the pre-incubation of cells *in vitro* (Table II). However, no inhibitory effect of chondro-4-sulphatase and chondro-6-sulphatase on the tumour growth was observed under any conditions (Table I).

*Effect of streptomyces hyaluronidase and β-glucuronidase.*—Streptomyces hyaluronidase and β-glucuronidase had no inhibitory effect on the tumour growth (Table I).

*Effect of chondroitin sulphates A, B and C.*—The injection of 1 ml of chondroitin sulphate A and condroitin sulphate C solution prior to tumour inoculation promoted tumour growth, whereas chondroitin sulphate B exhibited no growth-promoting effect (Table III).

### DISCUSSION

Present data indicate that chondroitinase-ABC and -AC inhibit the growth of solid Ehrlich ascites tumour, and that chondro-4-sulphatase, chondro-6-sulphatase, streptomyces hyaluronidase and β-glucuronidase have no inhibitory effect on tumour growth. Yamagata *et al.* (1968) determined that the optimal pH for the degradation of chondroitin sulphates A, B and C by purified preparation of chondroitinase-ABC was between 7·0–9·0. In this experiment there was no difference in the inhibitory effect of chondroitinase-ABC between Veronal buffer solution (pH 8·0) and saline solution of it (pH about 6·5).

Recently, Kojima and Maekawa (1970) and Kojima and Yamagata (1971) revealed that chondroitinase causes a reduction of electrophoretic mobility as well as the release of chondroitin sulphates from the surface of AH 130 cells; their data also demonstrated the presence of chondroitin sulphate A and chondroitin sulphate C at the surface of tumour cells. Suzuki *et al.* (1970), studying the effect of chondroitinase-treatment on the cell surface of HeLa-$S_3$ and L-929, demonstrated that chondroitin sulphates A and C and dermatan sulphate are major constituents of the cell surface and suggested that they play an important role in ensuring the normal surface character of the cells.

Ozzello, Lasfargeus and Murray (1960)

TABLE III.—*Effect of Chondroitin Sulphates A, B and C on the Growth of Solid Ehrlich Ascites Tumour on the 8th Day after Subcutaneous Inoculation in Mice*

| Chondroitin sulphate injected | No. of mice | Tumour size (controls ≡ 100) | Tumour weight (mg)† | P values* |
|---|---|---|---|---|
| None | 40 | — | 1081±72 | — |
| 2% Chondroitin sulphate A | 40 | 162 | 1751±86 | $P<0\cdot001$ |
| 2% Chondroitin sulphate B | 40 | 113 | 1218±64 | $0\cdot1<P<0\cdot2$ |
| 2% Chondroitin sulphate C | 40 | 169 | 1825±125 | $P<0\cdot001$ |

* *P* values designate the statistical significance of the difference in average tumour weight between experimental groups and control (saline) groups.
† Mean ±S.E.

ascribed the growth-promoting activity of acid mucopolysaccharides to the negative electric charge and the viscosity. Morrison *et al.* (1965), demonstrating the growth-stimulating action of small amounts of chondroitin sulphate A in several types of cells *in vitro*, considered that chondroitin sulphate may be a metabolic agent in the regulation of cell processes. Lippman (1968) described that treatment of Moloney-induced tumour with heparin and other acid mucopolysaccharides blocked cell surface antigens thereby altering transplantation behaviour.

The data in these experiments reveal that chondroitin sulphate A and chondroitin sulphate C stimulate tumour growth, and that chondroitinase-ABC and -AC inhibit the tumour growth *in vivo* and after pre-incubation *in vitro*, even though no difference in viability between the chondroitinase-ABC-treated cells and sham-treated cells was revealed by the neutral red exclusion test. From these results it is conceivable that chondroitin sulphate serves as a growth supporter which protects the surface of tumour cells and promotes the physiological surface function of the cells.

The author is grateful to Dr K. Kojima of the Aichi Cancer Centre Research Institute for his fruitful discussion, and to Dr M. Kodama of the Aichi Cancer Centre Research Institute for the supply of tumours.

### REFERENCES

KAZIWARA, K. (1954) Deviation of Stable Polyploid Sublines from a Hyperdiploid Ehrlich Ascites Carcinoma. *Cancer Res.*, **14**, 795.

KOJIMA, K. & MAEKAWA, A. (1970) Difference in Electrokinetic Charge of Cells between Two Cell Types of Ascites Hepatoma after Removal of Sialic Acid. *Cancer Res.*, **30**, 2858.

KOJIMA, K. & YAMAGATA, T. (1971) Glycosaminoglycans and Electro-kinetic Behaviour of Rat Ascites Hepatoma Cells. *Expl Cell Res.*, **67**, 142.

LIPPMAN, M. (1968) Transplantation and Cytotoxicity Changes Induced by Acid Mucopolysaccharides. *Nature, Lond.*, **219**, 33.

MORRISON, L. M., SCHJEIDE, O. A., QUILLIGAN, J. J., FREEMAN, L. & MURATA, K. (1965) Metabolic Parameters of the Growth-stimulating Effect of Chondroitin Sulfate A in Tissue Cultures. *Proc. Soc. exp. Biol. Med.*, **119**, 618.

OHYA, T. & KANEKO, Y. (1970) Novel Hyaluronidase from Streptomyces. *Biochim. biophys. Acta*, **198**, 607.

OZZELLO, L., LASFARGEUS, E. Y. & MURRAY, M. R. (1960) Growth-promoting Activity of Acid Mucopolysaccharides on a Strain of Human Mammary Carcinoma Cells. *Cancer Res.*, **20**, 600.

SUZUKI, S., KOJIMA, K. & UTSUMI, K. R. (1970) Production of Sulfated Mucopolysaccharides by Established Cell Lines of Fibroblastic and non-fibroblastic Origin. *Biochim. biophys. Acta*, **222**, 240.

SUZUKI, S., SAITO, H., YAMAGATA, T., ANNO, K., SENO, N., KAWAI, Y. & FURUHASHI, T. (1968) Formation of Three Types of Disulfated Disaccharides from Chondroitin Sulfates by Chondroitinase Digestion. *J. biol. Chem.*, **243**, 1543.

TAKEUCHI, J. (1965) Growth-promoting Effect of Chondroitin Sulphate on Solid Ehrlich Ascites Tumour. *Nature, Lond.*, **207**, 537.

TAKEUCHI, J. (1966*a*) Growth-promoting Effect of Acid Mucopolysaccharides on Ehrlich Ascites Tumor. *Cancer Res.*, **26**, 797.

TAKEUCHI, J. (1966*b*) Effect of Chondroitin Sulphate on the Growth of Solid Ehrlich Ascites Tumour under the Influence of Hydrocortisone. *Br. J. Cancer*, **20**, 847.

TAKEUCHI, J. (1968) Effect of Chondroitin Sulfate on the Growth of Solid Ehrlich Ascites Tumor under the Influences of other Interstitial Components. *Cancer Res.*, **28**, 1520.

TAKEUCHI, J. (1971) Experimental Study on Interaction between the Growth of Malignant Tumor and Connective Tissue with Special References to Acid Mucopolysaccharides. *Acta path. jap.*, **21**, 1.

YAMAGATA, T., SAITO, H., HABUCHI, O. & SUZUKI, S. (1968) Purification and Properties of Bacterial Chondroitinase and Chondrosulfatase. *J. biol. Chem.*, **243**, 1523.